# The pancreatic beta cell surface proteome

**DOI:** 10.1007/s00125-012-2531-3

**Published:** 2012-03-31

**Authors:** I. Stützer, D. Esterházy, M. Stoffel

**Affiliations:** 1 Institute of Molecular Systems Biology, HPT E73, ETH Zurich, Wolfgang-Pauli-Str. 16, 8093 Zurich, Switzerland; 2 Competence Center for Systems Physiology and Metabolic Diseases, ETH Zurich, Zurich, Switzerland; 3 Faculty of Medicine, University of Zurich, Zurich, Switzerland

**Keywords:** Biomarker, Islet, Pancreatic beta cell, Proteomics, Review, Secretome, Sheddome, Surface proteome

## Abstract

The pancreatic beta cell is responsible for maintaining normoglycaemia by secreting an appropriate amount of insulin according to blood glucose levels. The accurate sensing of the beta cell extracellular environment is therefore crucial to this endocrine function and is transmitted via its cell surface proteome. Various surface proteins that mediate or affect beta cell endocrine function have been identified, including growth factor and cytokine receptors, transporters, ion channels and proteases, attributing important roles to surface proteins in the adaptive behaviour of beta cells in response to acute and chronic environmental changes. However, the largely unknown composition of the beta cell surface proteome is likely to harbour yet more information about these mechanisms and provide novel points of therapeutic intervention and diagnostic tools. This article will provide an overview of the functional complexity of the beta cell surface proteome and selected surface proteins, outline the mechanisms by which their activity may be modulated, discuss the methods and challenges of comprehensively mapping and studying the beta cell surface proteome, and address the potential of this interesting subproteome for diagnostic and therapeutic applications in human disease.

**Electronic supplementary material:**

The online version of this article (doi:10.1007/s00125-012-2531-3) contains peer-reviewed but unedited supplementary material, which is available to authorised users.

## Introduction

The endocrine part of the pancreas is composed of the islets of Langerhans, which are highly vascularised and innervated ‘mini-organs’ accounting for 1–2% of the total pancreatic mass. An islet consists of 50–80% of insulin-secreting beta cells [1], which are clustered with four other endocrine cell types: the glucagon-secreting alpha cells, somatostatin-secreting delta cells, pancreatic polypeptide-secreting cells and ghrelin-producing epsilon cells. The endocrine function of the beta cell is to respond to increases in blood glucose levels by secreting insulin, which triggers the uptake of glucose from the blood in insulin-sensitive tissues, where glucose is used as a fuel for ATP generation or converted into energy-storing macromolecules. Additionally, insulin acts as a growth hormone and induces satiety signalling in the brain, again, indicating glucose availability. Transiently, beta cells respond to elevated glucose levels by increasing insulin secretion (and workload) per cell. Chronically augmented insulin demands, such as during pregnancy, child growth and insulin resistance in obesity, are responded to not only by increased insulin secretion, but also by beta cell proliferation, so that the net insulin secretory capacity is raised, the individual workload is reduced and the beta cells are protected from glucotoxicity [2]. These adaptive processes are collectively termed ‘beta cell compensation’ [3] and, in addition to raised blood glucose levels, require the communication of pancreatic beta cells with other tissues (via circulating factors) and neighbouring cells that may be endocrine or non-endocrine, e.g. of vascular, neuronal or haematopoietic origin [4].

In line with this notion, various surface proteins that are critical for beta cell function, in that they participate in many pivotal steps of glucose-stimulated insulin secretion (GSIS) and are essential for proliferation, islet integrity and differentiation, have been identified. With the increasing prevalence of diabetes [5], focused attention has also been given to the mechanisms that lead to beta cell dysfunction and deficits in beta cell mass in type 1 and type 2 diabetes. However, the list of well-characterised beta cell surface proteins is short and composed of individual members that have been studied in different laboratories in the context of the respective research focus. Thus, the systematic mapping and characterisation of the beta cell surface proteome may be crucial for better understanding beta cell physiology in health and disease-related abnormalities. Furthermore, from a practical point of view, surface proteins are potentially more accessible than intracellular targets and are therefore particularly interesting targets for the development of diagnostic and therapeutic strategies.

## The beta cell surface proteome inventory

The beta cell surface proteome is defined by the entire set of beta cell proteins that are either embedded in, or extracellularly associated with, the plasma membrane. Given that the beta cell is a secretory cell, the secretome, which is the collective set of proteins that is released by a cell through classical (signal peptide-dependent) and unconventional or exosomal pathways [6], is part of the surface proteome as well. Some proteins pass through the plasma membrane only during the exocytotic process, while others integrate through vesicle docking/fusion or associate with cell surface receptors after secretion. Another subset of the proteome is the sheddome, comprising the cell surface protein extracellular cleavage products that arise from proteolytic processing at the plasma membrane. Together with the secretome, the sheddome is potentially a clinically relevant source of biomarkers and therapeutic target discoveries, which will be described later.

**Fig. 1:**
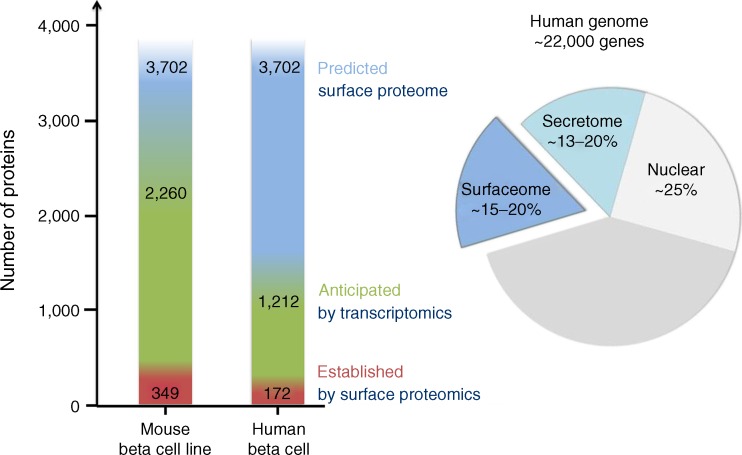
Chart of cell surface proteins predicted to be expressed in human beta cells. At 15–20 %, the predicted human surface membrane proteome constitutes a large part of the whole proteome [7, 8, 100]. Predicted cell surface proteins (blue) [7], anticipated cell surface proteins as determined by mRNA expression in human beta cells by the Beta Cell Gene Atlas (green) [9] and high-confidence human beta cell surface proteins as determined by N-linked glycoprotein cell surface proteomics (red) [10]. Note that for a large proportion of the predicted cell surface proteins no gene expression data are available. The secretome and sheddome were not considered in this graph because of a lack of comprehensive proteomic data

The total number and relative abundance of beta cell surface proteins is currently unknown, but computational prediction tools combined with transcriptomic and proteomic approaches can tell us what the beta cell surface may look like (Fig. 1). In a recent bioinformatics approach, a total of 3,702 transmembrane proteins were predicted, accounting for ∼15–20% of all human genes [7] (similar percentages are anticipated for the human secretome [8]). Searching for these proteins in the Beta Cell Gene Atlas [9]—a collection of publically available microarray data generated from the analysis of pancreatic beta cells and related cell types—reveals that, of these, 1,212 genes are expressed in primary human beta cells, while 353 are not (no data are available for the remainder; see Electronic supplementary material [ESM] Table 1). A recent proteomic study aimed at the identification of N-linked glycoproteins identified nearly 1,000 glycoproteins of mouse beta cells and human islets (available online via the dBETA database, http://biodata.ethz.ch/cgi-bin/beta.py), of which 349 proteins were found at the cell surface in the murine beta cell line MIN6 [10]. Of these, 172 proteins are also found in human islets, thus representing potential human beta cell surface proteins [10]. With further progress and refinements in mass spectrometry-based technologies, hypothetical beta cell surface candidate proteins (i.e. bioinformatically predicted surface proteins expressed in beta cells according to transcriptomic data) as well as possibly unanticipated surface proteins (which are, owing to the lack of a signal peptide, not predicted but may localise to the cell surface via unconventional pathways [11]) are likely to be validated or identified. In addition, it is likely that the beta cell surface displays proteins that are also produced in other, non-endocrine tissues, although possibly at different levels. However the beta cell plasma membrane proteome may be defined by beta cell-enriched macromolecules that are shared primarily with other neuroendocrine cell types or that are really beta cell specific. Several studies have indeed identified islet and beta cell enriched surface proteins [12–16]. Hence, comparisons of cell surface proteins between different cell types reveal specificity as well as the selective absence of proteins in beta cells. In fact, the concept of beta cell specific depletion of cell surface proteins has already emerged [17], which can contribute to the understanding of beta cell physiology as well. For example, expression of platelet-derived growth factor receptor declines over the course of a lifetime, which correlates with the age-dependent loss of beta cell proliferative capacity [18]; the lack of monocarboxylate transporter-1 explains the pyruvate paradox (i.e. the failure of pyruvate and lactate to stimulate insulin secretion), which, in turn, avoids inappropriate insulin release during physical exercise [19].

**Fig. 2:**
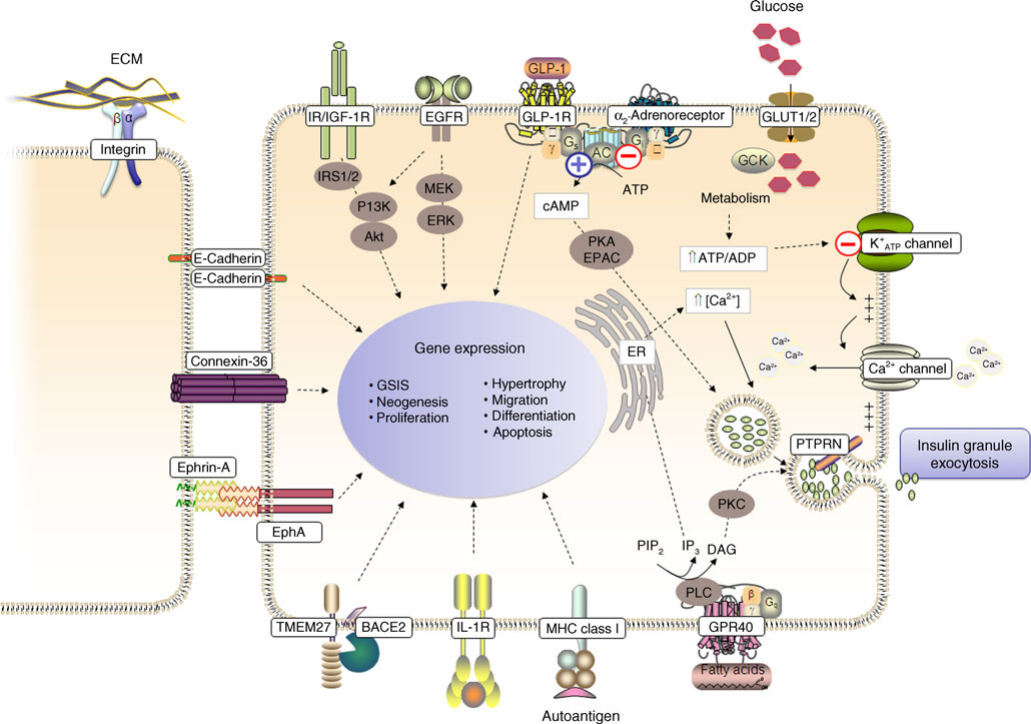
Selected beta cell surface proteins implicated in important functional aspects of the beta cell. Binding of insulin to the insulin receptor (IR) and insulin-like growth factor receptor (IGF-1R), as well as EGFR signalling, activates downstream effector molecules that positively regulate beta cell function and proliferation. In insulin granule exocytosis glucose is transported across the beta cell membrane via the glucose transporters GLUT2 and GLUT1 in humans. The metabolism of glucose results in a rise in the ATP/ADP ratio, which promotes ATP-sensitive potassium channel (K_ATP_ channel) closure, membrane depolarization, and opening of voltage-gated calcium channels. The increasing intracellular Ca^2+^ concentrations trigger the exocytosis of insulin granules. Insulin secretion is also influenced by fatty acids through G-protein coupled receptor (GPR40) signalling by the mobilisation of endoplasmic reticulum (ER) calcium stores and signalling through protein kinase C (PKC). GLP-1 receptor stimulates glucose induced insulin secretion by increasing cyclic adenosine 3′,5′-monophosphate (cAMP) levels and activation of protein kinase A (PKA) and guanine nucleotide exchange factor EPAC2. PKA and PKC can lead to phosphorylation of multiple proteins involved in the regulation of insulin secretion. In contrast, alpha adrenergic receptors inactivate adenyl cyclase (AC) and the formation of ATP, thus inhibiting insulin release. The solid lines indicate direct, immediate reactions. Note: The figure does not contain details of all the downstream signalling pathways of the depicted beta cell surface proteins. For further explanations, see text. DAG, diacylglycerol; IL-1R, interleukin-1 receptor; IRS, insulin receptor substrate; IP_3_, inositol 1,4,5-trisphosphate; MEK/ERK, mitogen-activated protein kinase/extracellular signal-regulated kinase kinase; MHC, major histocompatibility complex; PIP_2_, phosphatidylinositol 4,5-bisphosphate; PI3K, phosphatidylinositol 3-kinase; PLC, phospholipase C

The so far *functionally* studied surface proteome of pancreatic beta cells is composed of a structurally diverse network of proteins that interacts with environmental molecules, including metabolites, ions, hormones, various peptides and proteins (Fig. 2). According to Gene Ontology (GO) analysis the majority of its members fall into the categories of proteins with receptor, transporter, calcium ion binding, peptidase, kinase, G-protein coupled receptor and ion channel activity [10]. Most of them have also been linked to one of the three most important aspects in the life of a healthy beta cell, namely differentiation, survival/proliferation and insulin secretion. The function of some ‘classical’ beta cell surface receptors and their downstream signalling pathways, depicted in Fig. 2, have been extensively covered elsewhere [20–22], but in the context of mapping the beta cell surface proteome, special attention should also be paid to the proteins mediating cell–cell contacts: when working with primary islets and isolated beta cells their activity will have to be interrupted in most cases, and yet they are pivotal for beta cell function.

The concept that beta cells cannot operate properly autonomically but rather require intercellular contacts with other beta cells and surrounding endocrine and non-endocrine cells is underscored by early observations that isolated beta cells secrete less insulin in response to glucose compared with intact islets and that reaggregation of islet cells can partially restore these deficits [23, 24]. Similarly, islets and purified beta cells exhibit a better survival rate and secrete more insulin in response to glucose when plated on extracellular matrix (ECM) components rather than on standard culture-treated plastic dishes [25, 26]. The major proteins that mediate cell–cell interactions and contacts with the ECM are cell adhesion molecules (CAMs) such as cadherins, immunoglobulin CAMs, and integrins, and defects in beta cell function have been observed when there is a lack of proteins from each of these classes. For example, E-cadherin deficiency prevents the formation of MIN6 pseudoislets, i.e. spherical cell clusters that show improved stimulated insulin secretion compared with monolayers [27], while overexpression of the gene for E-cadherin (*Cdh1*) is linked to the limited proliferation rate of these assemblies [28]. Furthermore, a transgenic mouse model producing a dominant-negative form of E-cadherin that displaces wild-type E-cadherin and N-cadherin on the beta cell surface shows defects in the clustering of beta cells with other endocrine cells into islets during pancreatic development [29]. Likewise, a Ca^2+^-independent CAM of the immunoglobulin superfamily, neural cell adhesion molecule (N-CAM), is implicated in pancreatic islet morphogenesis [30, 31], and N-CAM-deficient mice exhibit impaired segregation of cells during organogenesis of pancreatic islets [30], and the adult mice have hyperinsulinaemia and impaired glucose tolerance because of enhanced basal insulin secretion and impaired insulin release at high glucose concentrations, respectively [32]. Integrin receptors, heterodimers of an α- and a β-integrin chain, establish contacts with the ECM of non-islet cells (e.g. endothelial cells) and are also required for beta cell function. The ligand binding and activation of integrin receptors enables contact to a microenvironment formed by endothelial cells, a vascular niche, which supplies beta cells with a vascular basement membrane that stimulates endocrine function and permits beta cell proliferation [33, 34]. Another type of cell-to-cell contact that enables the direct exchange of cytoplasmic ions, metabolites and other small signalling molecules is established by gap junction channels. These intercellular membrane channels are formed by two connexons, which are tubular structures of six connexins joining end-to-end over the extracellular space. Rodent and human beta cell gap junctions are formed by connexin-36 [35, 36], which coordinates calcium signalling and synchronisation of beta cells. Decreases in connexin-36 levels lead to defects in basal and glucose-stimulated insulin secretion [37, 38]. Beta-to-beta cell contact also enables bidirectional signalling, which is mediated by EphA receptor tyrosine kinases and ephrin-A ligands [39]. Under low glucose conditions, forward signalling by EphA inhibits insulin secretion, whereas under high glucose conditions, reverse signalling through ephrin-A ligands enhances insulin secretion [39]. Finally, neuronal parasympathetic innervations of islets can, for example, activate muscarinic acetylcholine receptors such as M3 in beta cells to promote GSIS [40]. In contrast, sympathetic innervation, e.g. signalling through α_2_-adrenoreceptors inhibits insulin secretion [41]. Another layer of complexity is added by the fact that beta cells within the islet, and islets themselves, are morphologically and functionally heterogeneous [42], which is also reflected in differential cell surface profiles and dynamics. For example, beta cell surface levels of CAMs differ from one cell to another, and their abundance correlates with the functional activity of beta cells in vitro [26, 43, 44].

While a plethora of information on the beta cell surface proteome is thus available, there are three major deficits in our current view. First, its dynamic nature tends to be under-studied. The qualitative and quantitative composition of any surface proteome will vary according to the extracellular and intracellular environment, which may, for example, reflect an organism’s developmental stage or nutritional status, or could be influenced by specific pathologies. Second, there is no high-coverage dataset available that identifies the surface proteins that are dysregulated and are involved in type 1 and type 2 diabetes. Third, its global coverage (i.e. the identification/validation of proteins on the beta cell plasma membrane) is still poor (see Fig. 1). These three aspects will be discussed in the following sections.
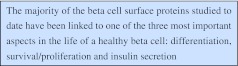


## Beta cell surface dynamics—transcription, traffic, turnover

Although some proteins are permanently expressed at the cell surface, the beta cell surface proteome is highly dynamic, with many factors being only temporarily exposed at the plasma membrane. In a constantly changing environment, the surface profile of a cell needs to be able to adapt appropriately. There are several mechanisms by which a cell may achieve this, and they include altered transcription, translation, protein trafficking, post-translational modifications (PTMs) and, in particular, selective and limited cleavage at the plasma membrane, which controls the fate and activity of various cell surface proteins. There may be a decision hierarchy to these processes, whereby gene expression tends to be the most long-term and long-lasting response, trafficking a faster adapting modulator, and certain PTMs the most acute reactions to change. Various plasma membrane proteins are subject to most of these pathways, which may act synergistically to ensure the tight regulation of surface expression.

Modulations in gene transcription in response to environmental changes have been observed for various beta cell surface proteins, one example being the expression of the prolactin receptor (*Prlr*) [45] and 5-hydroxytryptamine (serotonin) receptors 1D and 2B (*Htr1d* and *Htr2b*) [46]. Some high-coverage data on beta cell surface dynamics at the transcriptional level are probably hidden in various transcriptome-wide studies, but are subject to two pitfalls: (1) they only account for relatively long-term proteome changes, and (2) they do not always correlate with protein levels at the cell surface. Hence, they may not actually describe what the cell surface looks like, as the abundance of proteins in a cell is not necessarily uniform and localisation might be concentrated in specific, possibly non-plasma membrane compartments under a certain condition.

The transport of proteins to and from the plasma membrane is guided via vesicular trafficking between compartments of the secretory and endocytic pathways. Non-conventional secretory pathways have been described [11, 47] that may also contribute to the exposure of proteins on the beta cell surface. In the beta cell the insulin granule, which partially fuses with the plasma membrane during GSIS, is an additional source of proteins transiently exposed on the plasma membrane and so transmembrane proteins such as receptor-type tyrosine-protein phosphatase-like N (PTPRN), which are resident in insulin granules, are also found on the cell surface [48]. Over the years it has been shown that beta cells are polarised and that this asymmetry is reflected by the uneven distribution of surface proteins, such that the major glucose transporter, GLUT2, is enriched in lateral microvilli [49] or TMEM27, a mitogenic type1 transmembrane protein, is enriched in primary cilia [50]. Furthermore, specific surface proteins segregate in cholesterol-rich microdomains known as lipid rafts, such as the major beta cell voltage gated Ca^2+^ channel Ca_V_1.2 and the voltage-gated K^+^ channel K_V_2.1 [51]. Since these domains have been implicated in concentrating signal transduction modules and affecting important cellular functions such as GSIS [51–53], this selective targeting will also alter the function of the surfaceome. Again, however, comprehensive information on beta cell trafficking pathways and their cargo under specific conditions is currently lacking, not least because its acquisition requires challenging experimental set-ups and technologies that have only recently become available.

PTMs of surface proteins, including glycosylation, lipidation, sialylation, phosphorylation, sumoylation and acetylation, may also act as stabilising or destabilising signals. For example, glycosylation of GLUT2 is essential for its stability and proper localisation [54], and phosphorylation of epidermal growth factor receptor (EGFR) triggers its removal from the plasma membrane by endocytosis, a process known as receptor downregulation [55]. One particular form of PTM elicits turnover of cell surface proteins by proteolytic processing, which is of special interest for the following reasons: (1) as opposed to most other PTMs it is irreversible and thus a ‘stronger’ cellular decision; (2) it takes place at the plasma membrane itself, which exposes the process directly to the environment the cell needs to adapt to and makes it ideally poised for an imminent reaction; (3) given its cell surface location, it is potentially therapeutically accessible; and (4) the cleavage products may have biological functions on their own or, in theory, can be exploited as a readout for a particular cellular function(s). Ectodomain cleavage, or shedding, of membrane proteins is carried out by cell surface proteases, and this substrate-specific cleavage not only influences full-length protein activity, transport and turnover, but also generates a soluble and sometimes bioactive molecule. In fact, some shed fragments have been described to be functional soluble receptors or ligands, which participate in autocrine, juxtacrine and paracrine signalling, or act as decoy receptors that sequester ligands to permit permanent signalling [56]. They are evidently also potential biomarkers of beta cell function and mass (see later). In addition, the initial cleavage step by a sheddase also generates a membrane-bound fragment of a surface protein substrate, which is frequently subject to a further processing step termed regulated intramembrane proteolysis [56–58], which is thought to take place mainly in an intracellular compartment. The liberated intracellular cytosolic fragments may enter the nucleus to control gene transcription [56].

In recent years a growing number of transmembrane proteins of various topologies have been identified that are cleaved in the plane of the cellular membrane and, because of their diversity, they have been implicated in a number of different cellular processes, including cell signalling, cell adhesion, protein localisation, and pathological conditions such as cancer. On the flip side of the coin, several proteases have now been discovered, and the identification and functional characterisation of their substrate will likely determine which are the best candidates to be targeted by protease inhibitors. Of the roughly 650 proteases encoded in the murine genome [59, 60], about 130 predicted transmembrane or ECM-associated proteins are thought to be expressed in murine islets or beta cell lines (based on Affymetrix gene expression analysis). Of these, 29 have recently been confirmed to be expressed either by cell surface mass spectrometry or real-time PCR [15]. These include the classical sheddases, such as beta-site amyloid precursor protein cleaving enzymes 1 and 2 [BACE1 and BACE2] of the aspartyl protease family and members of the ‘a disintegrin and metalloproteinase’ (ADAM) family, as well as other proteases. Examples of beta cell-enriched cell surface protease–substrate pairs that regulate function and mass include ADAM10 and its substrate epidermal growth factor (EGF) that becomes a soluble ligand for EGFR and thus induces proliferation [61], the sheddase BACE2 and its target TMEM27, which is inactivated by cleavage [30], and the protease calpain 1, which cleaves PTPRN to produce an intracellular fragment that can enter the nucleus to stabilise active STAT5. Cleaved PTPRN thereby increases transcription of secretory granule genes and stimulates beta cell proliferation [62, 63]. However, this information is only a starting point for the study of the protease-specific sheddome and the regulation of the surface proteases themselves under different physiological and pathological conditions in beta cells.
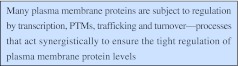


## The beta cell surface proteome in type 1 and type 2 diabetes

In the context of diabetes, there are two categories of beta cell surface proteins of interest: those that are causally involved in disease development and those that can be used for diagnostic and treatment purposes (of course, some proteins may fall into both classes). In healthy individuals the beta cell senses its environment and adjusts its function and mass to meet metabolic needs, ensuring that plasma glucose concentrations remain within a relatively narrow physiological range. Thus, insulin release by human beta cells can be increased by about four- to fivefold and beta cell mass enhanced by about 50% during insulin resistance [64, 65]. The beta cell surface proteins that contribute to type 1 diabetes are likely to be very different from those involved in type 2 diabetes. The fundamental pathology of type 1 diabetes is autoimmune-mediated beta cell destruction, which results in an absolute deficiency of insulin, with only about 1% of beta cell mass remaining in individuals with lifelong type 1 diabetes at autopsy [66, 67]. In contrast, type 2 diabetes is characterised by beta cell dysfunction that is thought to occur very early in the disease, even preceding insulin resistance [66, 68], and only later by reduced beta cell mass (a loss of up to 63% in long-term type 2 diabetes) [64]. Evidently, the systematic analysis of the changes in the beta cell surface proteome, sheddome and secretome in human diabetes and its development is challenging, but this is urgently required to gain a more comprehensive and molecular understanding of disease development and novel treatments.

Several type 1 diabetes susceptibility genes have been identified, with particular correlations found for histocompatibility antigens. HLA class I and class II genes present antigens to antigen-specific receptors that mediate antigen recognition by immune effectors. The chronic autoimmune reaction in type 1 diabetes is directed towards specific beta cell antigens, which include the beta cell surface proteins insulin, PTPRN (which is also known as insulinoma antigen 2 or IA-2) and zinc transporter 8 (also referred to as ZNT8 or SLC30A8) [69]. The presence of autoantibodies against these proteins is used for diagnostic purposes. Additionally, severe beta cell impairment and loss can be triggered by viruses that use beta cell surface proteins as receptors, e.g. the cell adhesion protein coxsackie virus and adenovirus receptor (CXADR) or Toll-like receptors. Indeed, virus genomes have been sequenced from samples obtained at the autopsy of individuals with type 1 diabetes [70] and virus-neutralising antibodies can be serologically detected in many type 1 diabetic patients [71], giving rise to the notion that beta cell surface protein-mediated viral infection can even be the triggering event for the disease. The viral infection may be cytotoxic on its own and/or cause the beta cell to increase its cell surface levels of the major histocompatibility complex, which presents beta cell proteins to T cells, and induce the beta cell secretome of inflammatory cytokines such as IFNα and IFNβ, which will also elicit an adaptive immune response (reviewed in detail elsewhere [72]).

Inflammation induced by the release of beta cell cytokines also plays a role in type 2 diabetes, but in this case it arises from endoplasmic reticulum stress caused by excessive nutrient uptake (collectively called glucotoxicity and lipotoxicity) and insulin production. Since the beta cell glucose transporter GLUT2 (and GLUT1 in humans) is constitutively expressed so that the cell can accurately sense blood glucose levels, beta cells may be particularly vulnerable to glucotoxicity. On the other hand, decreased islet GLUT2 levels have been found in a mouse model of muscle insulin resistance, along with reduced critical insulin granule surface proteins such as vesicle-associated membrane protein 2 (VAMP2) [73], suggesting that a vicious cycle of impaired glucose sensing and insulin secretion ensues over time. Another example of a cell surface protein that has even genetically been linked to type 2 diabetes is the fatty acid receptor GPR40 [74]. In addition, the beta cell secretome is altered in individuals with type 2 diabetes, for example, it displays increased amounts of proinsulin [75] and augmented levels of human islet amyloid polypeptide (IAPP) (which is co-secreted with insulin). Human IAPP, islet amyloid deposits of which are found in type 2 diabetes, aggregates in the ECM and induces apoptosis and defects in insulin secretion [76].

While the therapeutic potential of the beta cell surface will be discussed in detail later on, it is worth pointing out at this stage that many of the best-established treatments for diabetes simulate or boost the action of this subproteome. Insulin substitution is the only treatment available for type 1 diabetes at the moment. However, the hyperglycaemia in type 2 diabetes can be alleviated by sulfonylureas such as glibenclamide that bind and block the sulfonylurea receptor 1 (SUR1) subunits of the inwardly rectifying ATP-sensitive potassium channel (K_ATP_ channel), closure of which is essential for membrane depolarisation that initiates insulin secretion [77]. Dipeptidyl peptidase IV (DPPIV)-resistant glucagon-like peptide 1 (GLP-1) analogues (e.g. exenatide) or DPPIV inhibitors (e.g. sitagliptin) are used to potentiate GSIS and promote beta cell proliferation via increased GLP-1 receptor activity [20]. The relative success of the glucose-lowering strategies targeting the beta cell surface proteome highlight the importance of this subproteome in the disease.
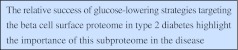


## Approaches to map and characterise the beta cell surface proteome

Clearly, the expansion of qualitative as well as quantitative, dynamic coverage of the pancreatic beta cell surface proteome will increase our understanding of this cell type. The logical approach is to (1) establish the plasma membrane proteins of a certain cell type under given conditions (finding the beta cell ‘bar code’); (2) distinguish between those crucial for the cell’s function and the dispensable/redundant ones, which is relevant for the identification of new therapeutic targets; and (3) selecting those that are somewhat cell specific and therefore confer target tissue selectivity. Mass spectrometry-based methods are the method of choice for the identification and quantification of proteins in complex protein samples [78]. While quantitative, dynamic proteome mapping is always challenging, this is particularly true in the case of the pancreatic beta cell surface proteome. Primary beta cells are harder to acquire than many other cell types (see below), and transmembrane proteins are the most challenging group of proteins to analyse as a result of the heterogenic and amphipathic nature of membrane proteins, combined with their limited abundance compared with cytosolic proteins. Thus, the largest determined (mouse) islet proteome of 6,873 proteins contains 952 islet-enriched proteins, about a third of which are categorised as membrane and extracellular proteins by GO analysis [79]. The limitations in the identification of surface proteins have been addressed, in part, by protocols that involve the enrichment and separation of membrane proteins from cytosolic proteins through subcellular fractionation or chemical labelling followed by mass spectrometric analysis [80]. Current methods encompass enriching centrifugation and extraction techniques [81], coating of plasma membranes using silica beads [82, 83], affinity-based approaches (e.g. lectin- and antibody-based protocols) [84–86], cell surface ‘shaving’ [87] of intact cells with proteases, and methods that chemically tag surface proteins such as biotinylation [88] and glycoprotein capturing [89–92]. A mixture of different approaches will ultimately provide higher coverage of the surface proteome, as illustrated by a recent study on human pancreatic islets in which a combination of different extraction techniques increased the total to 343 identified membrane proteins [93].

Chemical tagging strategies have been a popular choice for enrichment because labelled surface proteins can subsequently be resolved from unlabelled proteins by affinity purification, an advantage over classical ultracentrifugation preparations that are typically contaminated by proteins from intracellular membranes [80]. Plasma membrane biotinylation protocols involve the covalent labelling of extracellular proteins with a biotinylation reagent [94], followed by capture of the biotin-conjugated proteins or peptides via an avidin-/streptavidin-coated solid support. The bound proteins/peptides are eluted from the affinity matrix and analysed by mass spectrometry. Glycoprotein capturing is based on solid-phase extraction of N-linked glycopeptides, and has been successfully used to identify and quantify N-linked glycoproteins from serum and cellular samples [89, 91]. This method has been further developed by cell surface capturing (CSC), in which extracellular glycan moieties of intact, living cells are chemically labelled [90, 92]. Glycans on the surface of intact cells are oxidised and the formed aldehyde groups are coupled to a bi-functional linker molecule (biocytin hydrazide). After cell homogenisation, a crude membrane fraction is isolated by ultracentrifugation and proteins are extracted and digested. The labelled peptides are isolated using affinity capture, and the peptides are released by enzymatic cleavage of the carbohydrate chain and analysed by mass spectrometry. Both the whole cell glycocapture approach and the cell surface-capturing approach have been successfully used to identify beta cell N-linked glycoproteins, and quantitative monitoring identified 24 proteins that are up- or downregulated after stimulation by glucose and GLP-1 [10]. In addition, various strategies that are commonly used to determine surface protease substrates can also be applied to pancreatic beta cells. These involve the analysis of cell culture medium proteomes and the identification of the N-terminome, the amino acid sequence that is generated after cleavage of the amide bond by the protease [95]. Finally, protein candidates can be validated in follow-up studies using targeted proteomic approaches such as single reaction monitoring (SRM) assays, which enable the accurate quantification of selected peptides in complex samples [96].

The application of any of these proteomic techniques to primary pancreatic islets, and beta cells in particular, adds an extra hurdle to the task as, given the organ’s size and the ethical issues associated with the acquisition of primate material, the achievable input sample amounts are limited. The advantage of using primary purified beta cells is that contaminating proteins from other endocrine cell types, which bias the result, are removed. However, the disintegration of the unique islet structure followed by culture and cell sorting involves many treatments that affect cell viability and function (see above), which will reduce the functional integrity of the surface proteome. Therefore, the use of intact pancreatic islets in combination with insulinoma cell lines originating from different species and under different experimental conditions (e.g. high versus low glucose level media) facilitates the cataloguing of the beta cell surface proteome. In a complementary approach the surface proteome of beta cell-interacting cells, such as exocrine tissue and alpha cell lines, can be characterised to identify beta cell-specific versus contaminating/common proteins.
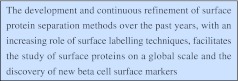


Although there is currently no ‘gold standard’ workflow that would allow the whole spectrum of the beta cell proteome to be covered, monitoring a critical number of beta cell surface proteins under different experimental perturbations (which should ideally be numerous and diverse) may be sufficient to unveil patterns of co- or antagonistic regulation, which could indicate common trafficking, localisation, interaction and function. This will accelerate the identification of putative therapeutic targets or biomarkers and at the same time provide a direction for the functional analysis of individual hits. Another crucial step in this initial assessment is the verification of whether the emerging candidate proteins are also expressed on human beta cell as, inevitably, the surface proteome may differ, especially quantitatively, between humans and other vertebrates. For example, it is known that rodent beta cells use GLUT2 for glucose uptake, while human beta cells express much less of this transporter and instead also take up glucose via GLUT1 [97]. Differences between humans and mice also exist in the expression of potential surface proteases [59] and, consequently, in substrate processing. The relevance of each cell surface protein for beta cell homeostasis can of course be tested in ‘classical’ functional beta cell assays using cell lines, primary islets and gain-/loss-of-function mouse models; however, the initial perturbation and pattern analysis from which the strongest candidates emerge may also hint at a more precise role of a protein, for example, a role in insulin granule recycling, establishment of cell polarity, fuel utilisation switching or specific nutrient uptake—all of which will, of course, if they are significantly altered, affect the fundamental clockwork of the beta cell.

## Biomedical applications of the beta cell surface landscape

**Fig. 3:**
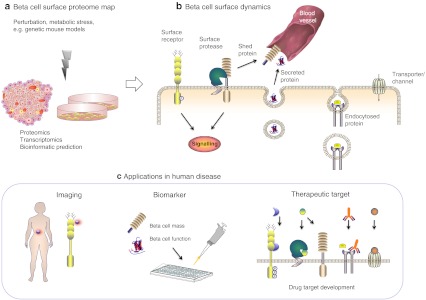
The three steps to characterising the beta cell surface proteome for diagnostic and therapeutic purposes. (**a**) Identification of the surface proteome, (**b**) analysis of its dynamic regulation, (**c**) assessment of the potential use of individual proteins as biomarkers, imaging tools and drug targets


What is the promise of a comprehensive beta cell surface map? The main hope is that it will be the gateway to targeting and monitoring the function and mass of a cell type that is scarce in number and otherwise hard to reach. The proteome will fall into two topological categories of useful proteins (Fig. 3): permanently beta cell membrane-associated proteins and shed/secreted proteins. The constitutive surface proteins may, provided they are beta cell-specific or -enriched, serve as drug targets and, in principle, as docking points/antigens for beta cell sorting and imaging. The advantage of targeting surface proteins is not only the likely accessibility of the proteins, but also the fact that very often they are found at the beginning of an amplifying signalling cascade, so that affecting one protein, such as a tyrosine kinase receptor, may have large and pleiotropic cellular effects. The current methods used to sort genetically unmodified beta cells (i.e. no fluorescent protein under a beta cell-specific promoter) are based on the differential autofluorescent properties of these cells compared with the rest of the islet as a result of different ion or metabolite contents [98], and unfortunately, yields, viability and purity are still poor. Imaging techniques, such as positron emission tomography (PET) and MRI can currently only reveal beta cells/islets that have previously been chemically treated ex vivo [99] because of the lack of a suitable antigen and/or antibody that could be coupled to a tracer and used non-invasively. The shed or secreted proteins may be used as indicators of beta cell mass, function or internal state in serum or urine. For example, the cleavage fragment of a structural component of beta cells will roughly correlate with the number of beta cells in the body. Such an accurate marker of beta cell mass is desperately needed to assess and appropriately treat type 1 and type 2 diabetes. At present, the main indicator is plasma insulin, levels of which can be maintained after a beta cell loss of up to 80% via functional compensation by the remaining pool. A stressed beta cell may secrete endoplasmic reticulum stress proteins or autophagosomal or apoptotic proteins that leak through the secretory pathway, and so the overall state of the beta cell could be evaluated. In a more therapy-specific way, the sheddome could also be used to monitor the effectiveness of a beta cell-selective protease inhibitor or islet regeneration/transplantation approaches. The challenge here is not only to find those molecules that are beta cell-enriched or beta cell-specific, but also those that are feasibly detectable by ELISA or other clinically compatible diagnostic techniques. Even without clearance by the kidney or other tissues, it is to be expected that such beta cell-derived proteins will be found in serum only at picograms per millilitre to low nanograms per millilitre levels—given that the concentrations of insulin, the most abundant secreted beta cell protein, lie in the nanogram range and, in comparison, prostate specific antigen (PSA), a secreted serine protease and marker for prostate cancer, is only present at low nanograms per millilitre levels even in cancer positive patients, yet is indicative of an organ that is more than 10 times larger than the entire beta cell pool (an average of 11 g prostate versus 0.8 g beta cells). However, if there is a need there is a way and, depending on the chosen biomarker, amplification methods may be at hand: for example, a hypothetical shed fragment with remaining enzymatic activity may be exploited to design an activity rather than concentration–based detection assay. The numerous potential applications of the beta cell derived surfaceome are thus, paradoxically, tightly linked not only to the importance of this cell but much rather precisely to its low abundance and anatomical remoteness.

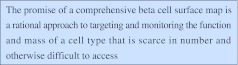


## Concluding remarks

Pancreatic beta cell functionality is crucial for maintaining glucose homeostasis. Historically, many proteins pivotal to its insulin secretory capacity and adaptability by mass expansion proved to be cell surface proteins, making the plasma membrane an attractive compartment for further gaining insight into how the beta cell senses and responds to its environment and for exploring candidates for drug development or beta cell markers. Also, early perturbing events at the cell surface could conceivably be involved in the development of beta cell dysfunction. Thus, qualitative and quantitative characterisation of the proteome may also help us understand these processes. With an increasing number of plasma membrane enrichment strategies followed by sensitive mass spectrometry, the dynamic surfaceome, sheddome and secretome of the beta cell are on the verge of being mapped. The challenges will then be to systematically assess these proteins functionally, and establish those that are critical members of the functional beta cell surface landscape and thus harbour therapeutic potential.

## Electronic supplementary material

Below is the link to the electronic supplementary material.

ESM Table 1 (XLSX 716 kb)

## Abbreviations

ADAM: A disintegrin and metalloproteinase
BACE2: Beta-site amyloid precursor protein cleaving enzyme 2
CAM: Cell adhesion molecule
ECM: Extracellular matrix
EGFR: Epidermal growth factor receptor
GO: Gene Ontology
GLP-1: Glucagon-like peptide 1
GSIS: Glucose-stimulated insulin secretion
IAPP: Islet amyloid polypeptide
PTM: Post-translational modification
PTPRN: Receptor-type tyrosine-protein phosphatase-like N
